# Therapeutic benefits of music-based synchronous finger tapping in Parkinson’s disease—an fNIRS study protocol for randomized controlled trial in Dalian, China

**DOI:** 10.1186/s13063-020-04770-9

**Published:** 2020-10-16

**Authors:** Lanlan Pu, Nauman Khalid Qureshi, Joanne Ly, Bingwei Zhang, Fengyu Cong, William C. Tang, Zhanhua Liang

**Affiliations:** 1 Department of Neurology, the First Affiliated Hospital of Dalian Medical University, Dalian, Liaoning Province China; 2 School of Biomedical Engineering, Faculty of Electronic Information and Electrical Engineering, Dalian University of Technology, Dalian, Liaoning Province China; 3 Department of Biomedical Engineering, University of California, Irvine, CA USA; 4 Faculty of Information Technology, University of Jyvaskyla, Jyvaskyla, Finland

**Keywords:** Music therapy, Parkinson’s disease, fNIRS, Randomized controlled trials, Explicit and implicit timing, Motor-control, Synchronous finger tapping

## Abstract

**Background:**

Music therapy improves neuronal activity and connectivity of healthy persons and patients with clinical symptoms of neurological diseases like Parkinson’s disease, Alzheimer’s disease, and major depression. Despite the plethora of publications that have reported the positive effects of music interventions, little is known about how music improves neuronal activity and connectivity in afflicted patients.

**Methods:**

For patients suffering from Parkinson’s disease (PD), we propose a daily 25-min music-based synchronous finger tapping (SFT) intervention for 8 weeks. Eligible participants with PD are split into two groups: an intervention group and a control arm. In addition, a third cohort of healthy controls will be recruited. Assessment of finger tapping performances, the Unified Parkinson’s Disease Rating Scale (UPDRS), an n-back test, the Montreal Cognitive Assessment (MoCA), as well as oxygenated hemoglobin (HbO_2_), deoxygenated hemoglobin (HbR), and total hemoglobin activation collected by functional near-infrared spectroscopy (fNIRS) are measured at baseline, week 4 (during), week 8 (post), and week 12 (retention) of the study. Data collected from the two PD groups are compared to baseline performances from healthy controls.

**Discussion:**

This exploratory prospective trial study investigates the cortical neuronal activity and therapeutic effects associated with an auditory external cue used to induce automatic and implicit synchronous finger tapping in patients diagnosed with PD. The extent to which the intervention is effective may be dependent on the severity of the disease. The study’s findings are used to inform larger clinical studies for optimization and further exploration of the therapeutic effects of movement-based music therapy on neural activity in neurological diseases.

**Trial registration:**

ClinicalTrials.gov NCT04212897. Registered on December 30, 2019. The participant recruitment and study protocol have received ethical approval from the First Affiliated Hospital of Dalian Medical University. The hospital Protocol Record number is PJ-KY-2019-123. The protocol was named “fNIRS Studies of Music Intervention of Parkinson’s Disease.” The current protocol is version 1.1, revised on September 1, 2020.

## Administrative information

**Table:** 

Title {1}	Therapeutic Benefits of Music-Based Synchronous Finger Tapping In Parkinson’s Disease – an fNIRS Study Protocol for Randomized Controlled Trial in Dalian, China
Trial registration {2a and 2b}.	Protocol Record number is PJ-KY-2019-123named “fNIRS Studies of Music Intervention of Parkinson's Disease.” WHO Trial Registration Data Set is not available. Trial was registered in Clinicaltrial.gov.
Protocol version {3}	Version 1.1 - September 1, 2020 with the Identifier of NCT04212897
Funding {4}	**Project title**: Program of Individualized Diagnosis and Treatment of Parkinson’s Disease and Related Dyskinesia and Construction of Neural Regulation Platform (LNCCC-C06-2015) **Awarding organization**: Health and Family Planning Commission of Liaoning Province, People’s Republic of China **Date of approval**: 1 January 2015 **Full name of the trial**: Program of Individualized Diagnosis and Treatment of Parkinson’s Disease and Related Dyskinesia and Construction of Neural Regulation Platform **Full name of the organization giving ethical approval:** Dalian Medical University The funding body did not participate in the study design.
Author details {5a}	^1^Department of Neurology, the First Affiliated Hospital of Dalian Medical University, Dalian, Liaoning Province, China ^2^School of Biomedical Engineering, Faculty of Electronic Information and Electrical Engineering, Dalian University of Technology, Dalian, Liaoning Province, China ^3^Department of Biomedical Engineering, University of California, Irvine, California, USA ^4^Faculty of Information Technology, University of Jyvaskyla, Jyvaskyla, Finland
Name and contact information for the trial sponsor {5b}	First Affiliated Hospital of the Dalian Medical University 222 Zhongshan Road, Dalian, Liaoning, China, 116011 E-mail: dyyyirb@163.com Phone: +86 0411-83635963
Role of sponsor {5c}	Sponsors did not have any role in designing the study protocol.

## Introduction

### Background and rationale {6a}

Parkinson’s disease (PD) is the second most common neurodegenerative disease that afflicts more than 10 million people worldwide. This disease is characterized by motor disturbances such as resting tremors, bradykinesia, hypokinesia, rigidity, gait disorder, and postural instability [1]. The hallmark tremor and locomotive impairments are caused by the absence of dopamine, typically produced by the substantia nigra (SN). Biologically, Lewy bodies are pathological hallmarks of PD and are hypothesized to be caused by degeneration of midbrain dopaminergic neurons and intraneuronal protein aggregation in surviving neurons. The impairment of the dopaminergic signaling of the SN to key timing brain regions is responsible for maintaining balance and complex muscle movement. The neuronal basis of the perception of timing is understood as neural entrainment, where neuronal activity is synchronized with an external or perceived rhythm (e.g., visual, tactile, or auditory) such that these external cues generate a temporal expectation which allows for the prediction of the next event [2].

Humans unconsciously align motor movement to extrinsic sensory modalities such as visual, tactile, or auditory. Everyday examples of neural entrainments include synchronizing with the footsteps of another person when walking side-by-side or finger and arm movement when listening to an isochronous auditory stimulus of a metronome [3–8]. Music-based therapies are particularly advantageous for treating PD symptoms because it has been shown to be beneficial for improving rigidity and gait, which some pharmaceutical and surgical treatments do not treat effectively [9–12]. A common music-based rehabilitation example is coupling auditory cues, like beats from rhythmic songs or a metronome, with steps [13]. Furthermore, Janzen et al. reports that the effects of priming finger taps to regular beats can also improve gait in PD [14]. These therapies rely on the natural phenomenon of neural entrainment by coupling motor movement to external cues which is also known as sensorimotor coupling.

There are two distinct timing systems associated with sensorimotor coupling. The first hypothesis proposes two paradigms: “automatic timing” and “cognitive controlled timing” [15–17]. The former is believed to be relevant in short intervals of sub-second ranges, where engagement of the cerebellum and basal ganglia are more likely to occur. In comparison, cognitive controlled timing has been hypothesized to be associated with longer timing intervals where conscious, cortical regions such as the prefrontal areas and the supplementary motor area (SMA) are activated [18]. Distinguishing between these two systems is an ongoing endeavor [19].

An alternative to automatic and controlled timing theory, “explicit” and “implicit” timing attempts to describe the neural network involved in producing movement with defined durations (explicitly) in comparison to coordinating or predicting movement in response to a temporally expected external event (implicitly). Explicit timing has been associated with neuronal recruitment in the premotor cortex, basal ganglia, cerebellum, and SMA while implicit is predominantly dependent on the cerebellum [20–22]. PD is diagnosed as a deficit in explicit timing where there are abnormalities in internally generated motor movements [17].

From both schools of thought, PD can be described as deficient in one pathway (cognitive and explicit) and preserved in the other (automatic and implicit). Automatic and implicit timing can be engaged with an external cue (e.g., auditory, tactile, or visual stimuli) to compensate for the loss of cognitive and explicit timing functions. These timing impairments are thought to affect an individual’s internal clock, producing asynchrony measured in temporal integration tasks such as synchronous tapping activities [23]. 2.5 s to 3 s inter-beat intervals were experimentally found to be the upper limit for accurate timekeeping in normal subjects [24]. In comparison, patients with PD generally have difficulty reproducing isochronous intervals; individuals with advanced stages of PD were found to have larger errors in faster beats and shorter interval thresholds [25].

Neuroimaging studies using functional magnetic resonance imaging (fMRI) and rhythmic stimuli have revealed increased neural activity coupling between brain regions associated with auditory processing and movement control such as the premotor cortex, SMA, cerebellum, and the basal ganglia [26, 27]. The beneficial effects from an external pacing stimulus for coupling movement and perceptual timing are thought to be mediated by the cerebello-thalamo-cortical network which has been previously associated with timing [28]. Thus, compensation of a dysfunctional basal ganglia timing system, as seen in a patient with PD’s circuitry, may also be facilitated by the same network. Additional regions that fMRI has revealed to be associated with the observed timing benefits of cueing include the putamen, temporal and parietal cortical areas [29]. Rhythmic auditory stimulation may not merely improve motor control during gait, but more generally maybe used to enhance performance in tasks involving perceptual and motor timing (e.g., finger tapping or duration discrimination).

Complementary to fMRI findings, functional near-infrared spectroscopy (fNIRS) reveals similar cortical neural activity. Mahoney et al. reported significantly higher prefrontal oxygenation in patients with PD during postural control [30]. In another study measuring freezing of gait with fNIRS, significant prefrontal and Brodmann area 10 HbO_2_ (oxygenated hemoglobin) activation only occurred when a turn was anticipated. In comparison, oxygenation did not change during unanticipated turns [31]. Both studies support the hypothesis that explicit motor control (when a turn is anticipated) is impaired while implicit motor control is preserved in patients with PD. In general, most of the research has established that there is an increased HbO_2_ activation in the frontal lobes of patients with PD when walking, particularly the prefrontal cortex and premotor cortices [30, 32, 33].

The use of fNIRS to assess neuronal response and its changes over time with music therapy as an intervention in patients with PD has not been explored yet. We take advantage of fNIRS’s high time resolution, locomotive freedom, and capabilities to detect neural activity in relevant motor control cortices as our primary quantitative measurement for investigating the neurophysiological effects and cortical neural activity changes from coupling motor movements with an external sensory cue. This protocol describes the implementation of an 8-week at-home finger tapping exercise while listening to a commonly known melody for participants with PD as a form of music therapy. In addition to fNIRS, various other assessments are used to determine and to track changes produced from the intervention over a 12-week period.

## Objectives {7}

This study’s primary objective is to investigate how external auditory stimuli coupled with motor movement affect motor synchronization and control in patients with PD to further understand how movement based music therapies or other forms of trained auditory cued movement impact the neural activity in key cortical timing brain regions. To achieve this, we propose the following objectives:

1. Determine neuronal activation and motor control performance in normal, healthy controls (HC) whose lifestyles and demographic are like those of the PD intervention group and PD control group.
2. Implement an 8-week intervention protocol that utilizes a familiar melody (chosen specifically for the intended population) paired with a simple, at-home finger tapping exercise randomized controlled PD intervention group.
3. Use fNIRS and motor control assessments at four time points collected throughout the study to assess how an external auditory stimulus (over time and repetition) coupled with motor movement affects motor synchronization and control in participants with PD. The collection time points are as follows: the first is collected after consent and allocation while the last 3 are sequentially collected 4-weeks apart from one another (i.e., 4 weeks, 8 weeks, and 12 weeks after the baseline measurement).
4. Investigate the effects of music therapy in PD by comparing the neuronal activity changes and motor control performances between PD control arm and PD experimental group.

### Trial design {8}

The study is a parallel two-group randomized controlled trial comparing motor timing performance of PD participants with and without music-based synchronous finger tapping training. In addition, data collected from a third arm, healthy controls, are assessed and serve as a normal, nonpathological baseline.

## Methods: participants, interventions, and outcomes

### Study setting {9}

The assessments and data collection are to be conducted in the First Affiliated Hospital of Dalian Medical University, China. This trial has received ethical approval from the hospital and is anticipated to be a 2-year study including recruitment, intervention delivery, assessments, and data analysis.

### Eligibility criteria {10}

Potential participants will be recruited from the Neurology Department of the First Affiliated Hospital of Dalian Medical University in China. Only individuals who meet the inclusion criteria will be invited to participate in the study.

There will be two populations participating in this study: one will be patients diagnosed with PD with intervention and the other without. A group of healthy participants will be recruited to serve as healthy controls (HC) that will provide the baseline assessment data to gage the severity of PDs in the patient populations.

The following are the inclusion criteria for participants with PD recruitment:

1. Aged 40–80 years old, both genders, and right-handed;
2. Clinical diagnosis of idiopathic PD according to the 2015 Movement Disorder Society (MDS) Clinical Diagnostic Criteria for Parkinson’s Disease;
3. Rated as stage I to II on the Hoehn and Yahr scale;
4. Score greater than 21 points on the Montreal Cognitive Assessment (MoCA);
5. Maintain a stable dosing of anti-PD or deep-brain stimulation (DBS) treatment throughout the duration of the study;
6. Able to travel to and participate in the data collection process.

Exclusion criteria for participants with PD recruitments include:

1. Individuals who do not meet the inclusion criteria;
2. Presence of significant hearing impairments;
3. Extensive previous musical training;
4. A history of any other neurological condition (i.e., Alzheimer’s disease, epilepsy, stroke) or psychiatric disorders (i.e., major depression, psychoses).

The HC group will consist of subjects recruited from hospital staff and students as well as students and faculty members from Dalian University of Technology. In addition, participants’ spouses will also be invited to participate. The HC group must have the following qualities to be included:

1. Aged 40–80 years older, both genders, and right-handed.

HC will be excluded along the same criteria that was established for the PD recruitment.

### Who will take informed consent? {26a}

A list of eligible participants and their spouses is generated from examining patient medical histories from the First Affiliated Hospital of Dalian Medical University. BZ and ZL are responsible for recruitment and screening of the eligible participants. LP will complete study enrollment along with obtaining informed consent. Solicitation to eligible participants who meet the established study criteria will be made through phone calls or during routine check-ups. Participants reached over the phone will be invited to the hospital for a physical examination, review the study details, address any questions, and sign the consent form. No later than 1 week after the consent form is signed, will the baseline measurements be taken, and further at-home instructions be provided. Consent will be collected using a short form provided in Simplified Chinese (or in other languages upon request). The form includes a description of the study, reasonably foreseeable risks or discomfort to the participant, and the rights of the participant, including withdrawal of participation at any time. The form may be signed via signature or thumb print. If necessary, legally authorized representatives may also provide consent. Oral interpretation of the consent form may be provided for illiterate participants. Translators involved in consent taking will also be asked to sign the form. A copy of the form will be provided (paper and/or electronically) to the participant upon signage for the participant to keep as a record.

### Additional consent provisions for collection and use of participant data and biological specimens {26b}

On the consent form, participants are asked if their data can be used should they choose to withdraw from the trial. Participants are also asked for permission for the research team to share relevant data with personnel from the associated universities taking part in the research or from regulatory authorities, where relevant. The trial does not involve collecting biological specimen for storage.

## Interventions

### Explanation for the choice of comparators {6b}

The PD intervention group is compared with the PD control group and a healthy control (HC) group. The PD control group is composed of participants diagnosed with PD and who meet the inclusion criteria. This PD control group is instructed to maintain the participants’ prescribed medication and care documented at the time of baseline assessment including any form of deep brain stimulation, if applicable. Any deviation from what was recorded at the time of taking baseline assessments are recorded during assessments and included when analyzing the data.

The second control group, the HCs, is composed of participants who meet the HC group inclusion criteria. This group predominantly consists of healthy spouses of the PD participants and individuals who share similar demographics to the participants diagnosed with PD. Measurements from the HCs are necessary because they serve as a normal, non-pathological benchmark, which has not been recorded before with this specific demographic and type of study paradigm. Furthermore, since spouses typically have similar lifestyles as their partners the measurements taken may help reduce the uncontrollable deviations due to dietary habits, living conditions, and environmental factors. HCs are asked to continue normal routine and must report any major deviations from baseline assessment routine (e.g., new medication to treat non-neurological illnesses that may affect motor movement or major injuries like head trauma). These deviations are noted for data analysis.

The neuronal activities of generating motor movement or processing a rhythmic external cue each should separately elicit measurable neuronal activation. However, when combined, a sensorimotor integration effect occurs such that motor movement is the result of a rhythmic external cue. Thus, the use of the HC and PD controls is to examine how movement coupled with an external sensory cue (a rhythmic auditory stimulus and in this study, music with rich rhythmic contents) impacts motor synchronization and control in PD.

### Intervention description {11a}

After the baseline measurements, participants in the intervention arm will be trained to do a rhythmic auditory synchronous finger tapping (SFT) intervention task at the hospital and asked to practice at home for a total of 25 min split between two 10-min sets with a 5-min break between the sets every day for 8 weeks. During training sessions participants must listen to the instrumental of a pre-selected well-known Chinese melody, i.e., 荷塘月色 (pinyin: “hé táng yuè sè”; English: “Moonlight over the Lotus Pond” by Phoenix Legend) whose melody duration is 259 s. This song was selected for its strong beat and familiarity to most of the participants. Participants must follow the beats of the melody and tap their right index finger simultaneously to the beats. The training session will be conducted once daily after patients have taken their medication. They are also instructed to take a video recording of themselves with their smartphones for the entire duration of the training. They may also solicit the help of their primary caregivers for the recording. This is for the purpose of monitoring protocol compliance.

### Criteria for discontinuing or modifying allocated interventions {11b}

Participants may stop participation at any time during weeks 0–12 due to various reasons including failure to meet inclusion criteria or inability to continue at-home intervention.

### Strategies to improve adherence to interventions {11c}

Participants’ primary caregivers are asked to monitor and, if needed, record completion of sessions using a smartphone with video and audio recording capabilities. The videos will be collected weekly either digitally through a secure cloud server or transferred from the phone if participant is available to come to the hospital on a weekly basis. Participants who do not have caregivers may be provided with a phone ring stand to help with recording themselves, if needed. In addition, a phone with video and audio recording capabilities is provided for participants who do not have one. These videos will be reviewed by a research assistant who is not involved in data collection or enrollment. For the intervention group, videos are scored separately for adherence and accuracy. Accuracy is determined by matching the time sequence of the song to that of the one used during the assessment. The HC and the control arm’s videos are scored on a binary adherence score. These scores are manually inputted into a designated spreadsheet and updated in the database weekly.

### Relevant concomitant care permitted or prohibited during the trial {11d}

Participants must maintain a stable dosing of anti-PD or deep-brain stimulation (DBS) treatment throughout the duration of the study, if applicable to the individual.

### Provisions for post-trial care {30}

Not applicable to study; no harm is anticipated from this study as the equipment used has minimal risks.

### Outcomes {12}

The primary outcome measurements of this study are the following:

1. Improvement or preservation of SFT and continuous finger tapping (CFT) performances from baseline measurements for intervention group.
2. Preserved or improved the Unified Parkinson’s Disease Rating Scale (UPDRS) scores from baselines measurements for intervention group in comparison to control arm.
3. Reduced HbO_2_ levels in prefrontal cortex from baseline measurements for intervention group. Preserved or improved n-back task scores from baseline measurements for intervention group in comparison to control arm.
4. Preserved or improved Montreal Cognitive Assessment (MoCA) scores from baseline measurements for intervention group in comparison to control arm.

Secondary outcome measurements of the study include:

1. Preserved motor control performances, UPDRS scores, HbO_2_ levels, n-back task scores and MoCA scores from baseline measurements in healthy control group.
2. Preserved or aggravated motor control performances, UPDRS scores, HbO_2_ levels, n-back task scores, and MoCA scores from baseline measurements in PD control group.

These outcomes are determined by analyzing the differences between participants’ pre-, 4-week (during), 8-week (post), and 12-week (retention), neuroimaging, neurophysiological, and SFT performance scores as well as compared with the data from the control arms. Individual and grouped averaged measurements are examined.

Music-based therapies often report beneficial and positive results; however, little is known about how music improves neuronal activity and connectivity in individuals with neurological disease. The primary outcome measurements of this protocol: SFT and CFT performances, HbO2, UPDRS scores, n-back, and MoCA scores act as assessments to quantify the effects of a music-based synchronous finger tapping training in individuals with PD. The trial’s outcomes may be used to enhance current clinical care of patients with PD by supplementing or augmenting current PD treatments with music-based exercises. There are currently no anticipated harmful outcomes because the use of fNIRS has been shown to be clinically safe and feasible. Consequently, this study was determined to be a low-risk intervention by the participating hospital’s Ethics Committee and the Steering Committee.

### Participant timeline {13}

**Fig. 1:**
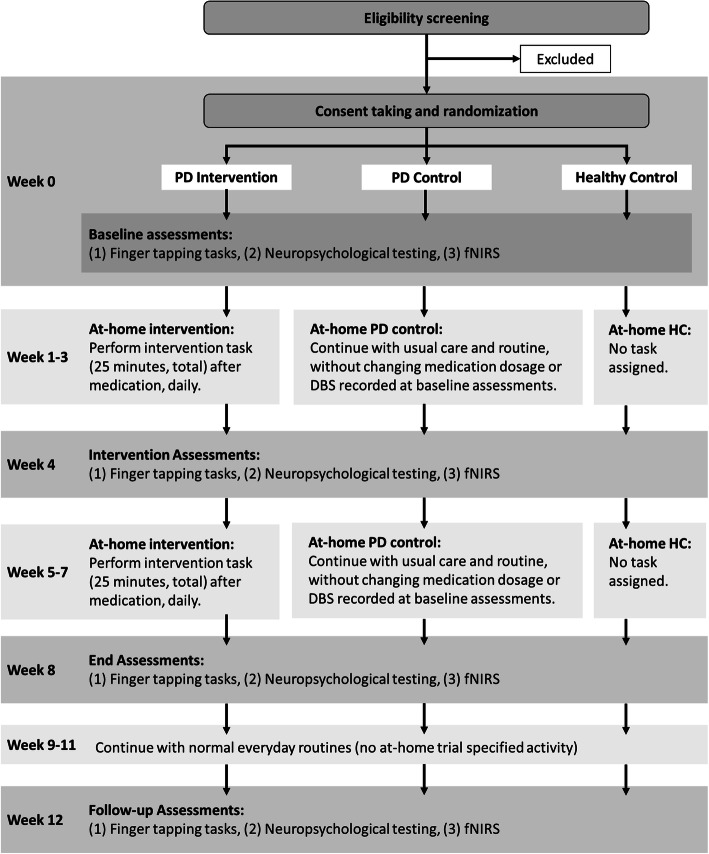
Expected trial plan. Not included in the diagram are excluded participants that may stop participation at any time during weeks 0–12 due to various reasons including failure to meet inclusion criteria or inability to continue at-home intervention. The intervention tasks are 25 min total, with two 10-min sets with a 5-min break between each set

**Fig. 2:**
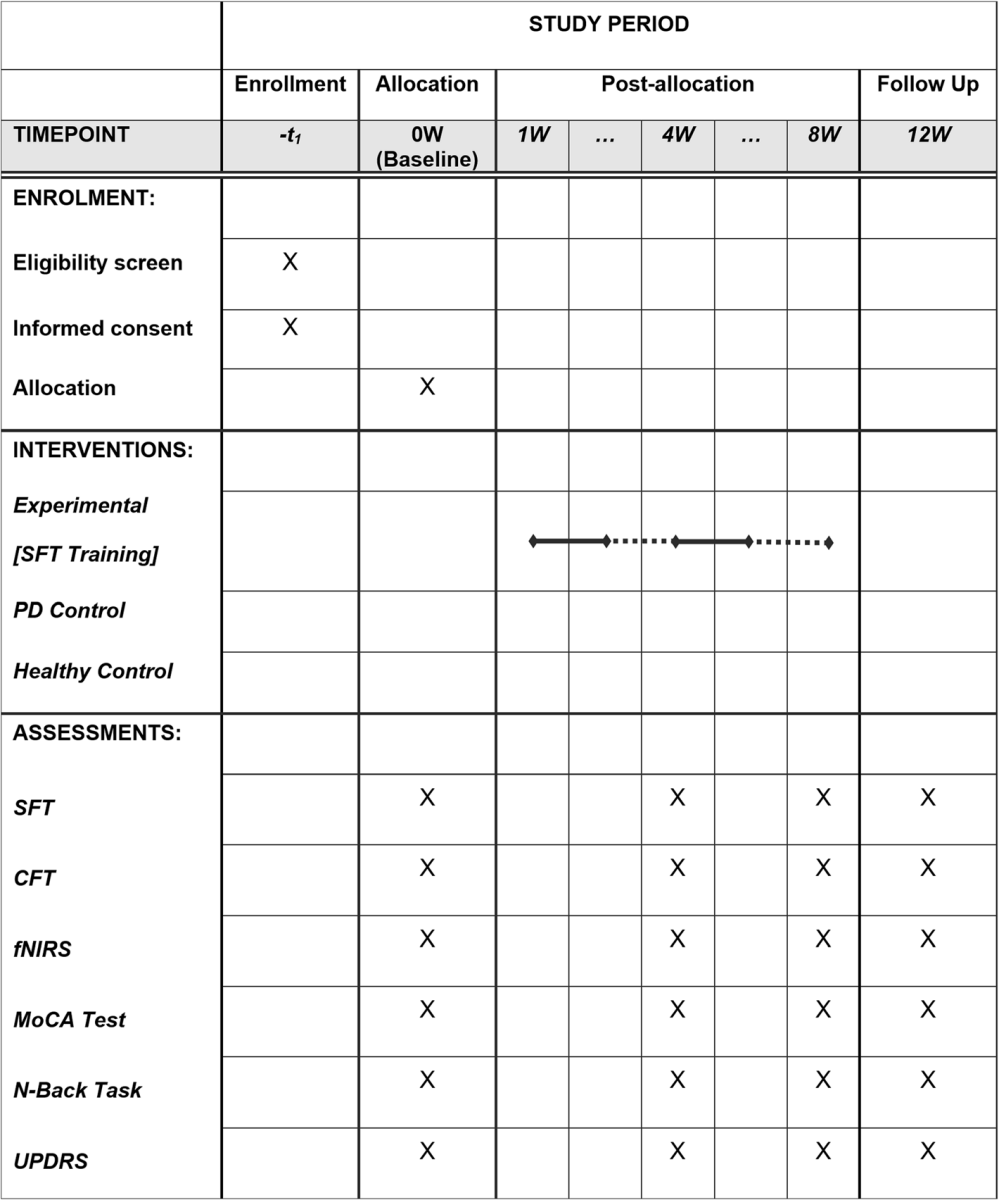
Standard Protocol Items: Recommendation for Interventional Trials (SPIRIT). W, weeks; SFT, synchronous finger tapping; HC, healthy control; CFT, continuous finger tapping; fNIRS, functional near-infrared spectroscopy; MoCA, Montreal Cognitive Assessment Test; UPDRS, Unified Parkinson’s Disease Rating Scale

Figure 1 graphically shows the participant timeline. Figure 2 illustrates the standard protocol.

### Sample size {14}

For this study, a total 210 participants (70 participants in three groups with a 10% attrition rate), are recruited over an 18-month period. We aim to recruit an even distribution of age, PD staged I, and II as determined by the Hoehn and Yahr scale, and gender to maximize statistical significance. This study is an exploratory prospective randomized controlled study with the primary outcome being the HbO_2_ value measured by fNIRS. Because there are no pilot study nor previous studies identical to this, the sample size was calculated by using power calculations (where *α* = 0.05, *β* = 80%, and we assume a conservative average difference between the PD groups). Klempir et al.’s study was used to estimate the baseline averages of HbO_2_ changes; however, there are notable inherent differences between this protocol and the referenced study. Klempir et al. describes HbO_2_ changes between participants with and without DBS during a finger tapping exercise [34]. Although our study may involve participants who use DBS as a treatment, we are not examining how various forms of treatment (pharmaceutical or DBS) affect motor control. Thus, the estimated HbO_2_ baselines when DBS is ON from Klempir’s study were approximations of the PD control group’s performance.

### Recruitment {15}

Potential participants are recruited from the First Affiliated Hospital of Dalian Medical University. Recruitment will be managed by BZ and ZL. Participants’ spouses are also invited to participate as healthy controls if they meet the inclusion criteria. We will assume a conservative estimate of 10% attrition due to various reasons such as voluntary withdrawal, illnesses during studies, and other uncontrolled factors.

## Assignment of interventions: allocation

### Sequence generation {16a}

Participants with PD are sorted into and then randomized into the intervention or control groups in a 1:1 balanced ratio stratified by gender and PD staged I and staged II as determined by the Hoehn and Yahr scale. Allocation into randomized study groups are conducted via a computer-generated random number table and determined during the pre-study procedures during allocation when informed consent and baseline measurements are made. The number generation is facilitated by R3.6.3, a computer random number generator typically used by the Scientific Research Department of the hospital. Participant groups are not known to clinical investigators, site personnel, nor data analyzers.

### Concealment mechanism {16b}

Once the consent form is received, a computer-generated random number from 1 to 210 will be assigned to that participant. Each number from 1 to 210 will be assigned a random group type: PD intervention, PD control, or HC. HCs can only be assigned numbers assigned an HC group type. For the numbers randomly allotted for PD participants, the computer-generated random number assignment will be set such that results in a 1:1 ratio, stratified by gender and PD staged I and staged II determined by the Hoehn and Yahr scale. The participant’s number will become the identifier for the participant’s collected data for data analysis. Once all participants have been enrolled and assigned, the list of which participants were distributed among the groups will be kept in an opaque, sealed envelope. Upon completion of consent, randomization, and baseline data collection, the participant will receive an opaque and sealed envelope, associated with their randomized number. The package will provide further at-home instructions as a physical copy, contact information for additional questions, and how to access a digital copy of their assigned group’s instructions. Verbal instructions, additional resources, and training for at-home tasks will be provided to participants and caregivers. Access to video instructions for at-home tasks is also provided as a supplement.

### Implementation {16c}

BZ and ZL will generate the allocation sequence and prepare the opaque envelopes with further instructions. LP will enroll participants, allocate participants, train participants and their caregivers for at-home tasks, and provide additional support to participants, accessible through phone calls, to ensure adherence.

## Assignment of interventions: blinding

### Who will be blinded {17a}

Primary investigators will be blinded to the data collected.

### Procedure for unblinding if needed {17b}

Not applicable to study; unblinding will not be utilized.

## Data collection and management

### Plans for assessment and collection of outcomes {18a}

Neuroimaging assessment: Functional near-infrared spectroscopy (fNIRS) will be employed to examine the cortical neural activity changes from the SFT intervention. The probe of the continuous wave system (ETG-4000, Hitachi Medical Co., Japan) will be used to measure changes in oxygenated (HbO_2_) and deoxygenated (HbR) hemoglobin [35, 36].

The 52-channel probe set will be placed on the prefrontal, premotor, and supplementary motor areas guided by the nerve navigation system and international 10–20 system for electrocochleography. The CZ and FZ positions from the 10–20 system are first identified. The central sulcus through CZ will be marked with a line and a second line, parallel to the first, will run through FZ. A third line, parallel and at the mid-point between the first two marks, will be used to align the midline of emitters-detectors. There are 20 emitters, set at 690 nm and 830 nm, respectively, and 16 detectors in the fNIRS head band, and will be used in a 52-channel configuration with each channel sampled at 10 Hz. The separation between each emitter and detector pair will be 3 cm [37, 38].

fNIRS will be taken at baseline, week 4, week 8 (end) and week 12 (follow-up) during motor-timing control assessments. Neuroimaging will be conducted for both the control and intervention arms. The data collected from this assessment is recorded digitally and uploaded to a designated cloud server immediately after assessment.

#### Motor-timing control assessment

A synchronous finger tapping assessment task will be used to measure participant’s motor-timing control. During the assessment fNIRS will be used to measure cortical neural activity. Participants will sit in front of a computer screen and asked to tap the “1” key, on the number pad of a standard QWERTY keyboard, as soon as the visual cue, a white star on a black background “ ” appears on the screen. This visual cue will appear for set durations. This duration will be 500 ms initially and will be adjusted based on the feedbacks from the subjects. SFT performance is scored by measuring the delay time between the cue for tapping and the recorded keystroke delivered by the participant. Delivery of the assessment and data collection will be facilitated by E-Prime 3.0, a computerized environment for psychological types of experimental design, data collection, and analysis. The performance in this assessment is recorded digitally and uploaded to a designated cloud server immediately after assessment.

**Fig. 3:**
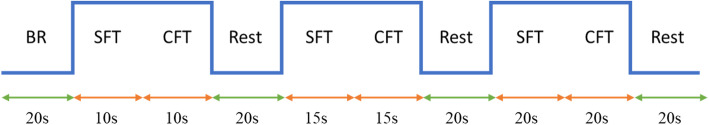
Schematic of experimental paradigm. BR, baseline rest; SFT, synchronous finger tapping; CFT, continuous finger tapping. In the SFT task, visual stimuli will appear at three different rates 1000 ms, 1500 ms, and 2000 ms respectively

The participants will be seated in a quiet room in front of a computer monitor. The fNIRS system and earphones will be fitted onto the participants and the audio’s volume will be adjusted to a comfortable level. Prior to assessment, the participants will be read instructions and perform a training module to ensure participants understand and know what to expect. Participants will be asked to relax and to restrict their motor motions before the start of the experimental paradigm. Subjects rest for the first 20 s to provide a fNIRS baseline signal correction. The participant will then be asked to complete the following tasks 3 times with 120 s break in between each 170 s set of the following:

1. Synchronous finger tapping (SFT): Subjects must tap right index finger as a visual cue appears. The visual cue will appear 10 times at the rate of 1000 ms, 1500 ms, and 2000 ms, respectively for every cycle. The number of times and rate the visual cue appear will be adjusted if no significant difference in learning is observed between patients with PD and healthy subjects.
2. Continuous finger tapping (CFT): Subjects are asked to tap their right index finger such that it maintains the previous temporal rhythm.
3. Rest: participants are asked to restrict their body movements (Fig. 3).

Three neuropsychological assessments will be employed for measuring the therapeutic effects of the music therapy on the disease. The Unified Parkinson’s Disease Rating Scale (UPDRS), n-back task, and Montreal Cognitive Assessment (MoCA) will also be measured at baseline, 4, 8, and 12 weeks.

Three neuropsychological assessments will be employed for measuring the therapeutic effects of the music therapy on the disease. The Unified Parkinson’s Disease Rating Scale (UPDRS), n-back task, and Montreal Cognitive Assessment (MoCA) will also be measured at baseline, 4, 8, and 12 weeks.

#### UPDRS

The UPDRS objectively assesses the severity of PD based off the disease’s burden on the individual and can describe disease progression and treatment response. A total of 42 ratings are split between multiple categories. Examples of categories measured include mental impairments (mood and intelligence), activities in daily living (speech, salivation, level of independence to perform normal tasks such as turning in bed), motor skills (facial, tremor severity in extremities, rigidity), and other complications. Each category has a 0–4 rating determined by the examiner and summed, where a higher score reflects greater disability (maxed at 195 points) [39]. The performance in this assessment is recorded digitally and uploaded to a designated cloud server immediately after assessment. If paper copies are used, then the document will be scanned and a digital copy with manual inputs will be made.

#### N-back task

The n-back task examines an individual’s working memory and working memory capacity by employing a visuo-spatial continuous performance task. The task used in this study was based on the n-back tasks from Beato et al. [40]. This study will employ 3-back tasks that are delivered digitally and started with a digital countdown. The participant is asked to input a keystroke on standard QWERTY keyboard with their right-hand to indicate whether a target visual stimulus presented on the screen was identical to a previously shown stimulus presented “n” trials ago. The stimulus will be a white square randomized into one of six positions on a black screen. The first cue stimulus will be presented on the screen for 3 s and the individual has 3 s to respond with a “1” keystroke to indicate “same” or a “2” keystroke to indicate “different.” A new stimulus will appear after a 1-s inter-stimulus interval. Each task will include responses to two sets of 15 and thus each assessment will last approximately 10 min. The performance in this assessment is recorded digitally and uploaded to a designated cloud server immediately after assessment.

#### MoCA

MoCA assesses for an individual’s cognitive abilities via a one-page, 30-point exam, delivered as a 10-min assessment. This includes orientation (6 points), concentration and attention (6 points), executive functions (3 points), memory (5 points), conceptual thinking (2 points), language (3 points), and visuo-constructional skills (5 points) [41]. The Chinese (Mandarin) MoCA readily available from mocatest.org will be utilized throughout this study [42]. The performance in this assessment is recorded digitally and uploaded to a designated cloud server immediately after assessment. If paper copies are used, then the document will be scanned and a digital copy with manual inputs will be made.

### Plans to promote participant retention and complete follow-up {18b}

Participants and participant family members will be given a reminder (via e-mail and phone call) a week before to schedule the follow-up.

### Data management {19}

All participant data will be identified as their trial identification number assigned during allocation. Each trial identification number will have their own designated folders. Data collected from fNIRS, motor control assessment, and N-back tests are conducted on a single laptop and will be automatically uploaded to their respective designated cloud storage databases immediately after each individual measurement. UPDRS and MoCA assessments will be recorded digitally using a digital copy of the assessments and saved into their respective participant folders. If paper copies are used, then the physical copies will be scanned and manually converted to a digital copy after the assessment. Any physical copies collected throughout the study will be stored for up to 5 years. A master datasheet that collects all digital data scores and is organized by participant number and time point will be pre-programmed to conduct range checks for all data values collected.

Video and audio recorded from participant phones or cameras will be uploaded onto the same database but stored separately from the data collected from assessments such that it is not accessible by data analysts or primary investigators. Only research assistants who are designated to transfer the files into the cloud storage or are trained to score the videos have access to the video and audio files.

### Confidentiality {27}

Confidentiality is not an issue in the data collecting country; however, the data will be stored on the cloud server provided by the hospital which is already secured to ensure strict access. Only primary researchers who are enrolling participants, collecting or analyzing data and other principal members of the research team will be able to access the cloud database. Before the data is uploaded to the cloud, it will be organized based on trial identification number instead of group and individuals’ names. Access to the database may be granted upon request from other researchers, study entities, or miscellaneous government associated groups who wish to conduct prospective meta-analyses. For international entities that request for data access, video recordings are pre-processed to de-identify the participants.

### Plans for collection, laboratory evaluation, and storage of biological specimens for genetic or molecular analysis in this trial/future use {33}

Not applicable to study; no biological specimens collected.

## Statistical methods

### Statistical methods for primary and secondary outcomes {20a}

Data analysis will commence after all retained recruited participants have completed their 12-week assessment.

#### Finger tapping performances and neuropsychological assessments

The SFT and CFT performances as well as neuropsychological measurements collected from the three groups (intervention and control arms) during assessment will be compared using a two-sample *t* test. Correlative data and the extent of the intervention’s effect on finger tapping performances and PD symptoms may be extrapolated from this data.

#### fNIRS data

An initial band pass filter will be applied to the raw fNIRS data before further processing to remove noise and drifts. Neuroactivity in various regions of interest including, prefrontal, pre-motor, supplementary, and primary cortex, will be analyzed by constructing a general linear model for each subject then estimating parameters and obtaining contrast values of the total oxy-Hb and deoxy-Hb signals. The contrast value images can be averaged within groups and a group analysis can be done using a *t* test. The averaged signal levels of each group will be plotted on a time vs total blood flow graph and compared to determine intervention effect.

### Interim analyses {21b}

Not applicable to study, data analysis is not anticipated until the end of studies (follow-up) for all participants.

### Methods for additional analyses (e.g., subgroup analyses) {20b}

Further statistical analysis may be explored to account for interacting factors including gender, age, and different stages of PD as determined by the Hoehn and Yahr scale.

### Methods in analysis to handle protocol non-adherence and any statistical methods to handle missing data {20c}

Missing data and any data collected not adhering to the protocols will be removed from data analysis.

### Plans to give access to the full protocol, participant level-data and statistical code {31c}

The datasets analyzed during the current study are available from the corresponding author on reasonable request.

## Oversight and monitoring

### Composition of the coordinating center and trial steering committee {5d}

The roles of the principal investigators (FC, WT, BZ, and ZL), research physicians (LP), and associated researchers (NKQ, JL) are to advise and agree upon protocols, organize steering committee meetings every other month, lead publications of study reports, and report updates to sponsors and key personnel of the Scientific Research Department of the hospital. The Steering Committee, chaired by ZL, is responsible for agreeing on the final protocol, recruiting and screening of participants, and reviewing the progress of the study to advise changes to the protocol and ensure smooth running of the study. The Trial Manager, LP, is responsible for the trial’s master file, obtaining approval from the Ethics Committee, coordinating audits with the Ethics Committee every quarter, managing enrollment and allocation, releasing official changes of clinical trial protocol to relevant entities and trial participants, scheduling participant assessments, reporting any serious unexpected adverse events, and addressing any questions participants have.

### Composition of the data monitoring committee, its role and reporting structure {21a}

The data collected is predominantly electronic and should be uploaded or manually inputted into the cloud-based storage server via a web interface. The hospital’s Ethics Committee and the study’s Steering Committee have determined that the intervention is considered “low-risk”; therefore, there is no formal data monitoring committee. Instead, the data is be managed by a designated Data Manager, LP, who oversees data recording, provides training to research assistants, and addresses any issues that arise from the cloud storage, data collection, data management, and data access. LP will frequently check and assess that data is collected accurately and reliably. Research assistants report to LP for additional assistance and guidance with the trial’s associated technologies, should issues arise. Any serious unexpected adverse events are expected to be reported to the data manager and thusly to the Steering Committee within 24 h.

### Adverse event reporting and harms {22}

The ETG-4000 fNIRS system has minimal risk. The audio is adjusted to a comfortable level prior to assessment. Consequently, no adverse event reporting plans are applicable to this study. Should an unforeseen serious adverse event occur, a case report is written, and the Trial Manager is notified within 24 h. The case report is expected to be shared with the Steering Committee, Ethic Committee, and trial participants within the next 48 h.

### Frequency and plans for auditing trial conduct {23}

The Ethics Committee of the hospital conducts audits quarterly (typically every 3–6 months) to check on progress and ensure adherence to the established protocol. If the study requires additional time beyond its previously determined set timeline, then a re-application to obtain permission for study continuation of the study is required. If this is necessary, then the Trial Manager is responsible for re-applying and obtaining committee approval.

In addition, the Steering Committee has scheduled meetings and check-ins every other month between the audits from the hospital’s Ethics Committee. This meeting is used to inform investigators, research physicians, and sponsors of updates, issues, and necessary changes to the study to be reviewed and agreed upon.

### Plans for communicating important protocol amendments to relevant parties (e.g., trial participants, ethical committees) {25}

Protocol amendments are first discussed with the Steering Committee during organized meeting times and agreed upon prior to formal release to the Ethical Committee and Scientific Research Department of the hospital. Once approval is obtained, a formal document summarizing the necessary protocol amendments are shared with relevant parties, especially trial participants via phone call and mail, with digital copies available upon request. Necessary updates to the clinical trial application are made by the Trial Manager upon approval from the Steering Committee, Scientific Research Department and the Ethics Committee. Should there be any deviations in the protocol, a protocol breach report will be completed and reported to the Trial Manager within 24 h. Data collected will be marked and considered during data analysis.

### Dissemination plans {31a}

Trial results are planned to be predominantly communicated via conferences and manuscript publication in relevant PD and fNIRS related organizations. A formal summary of the study’s results is provided to sponsors who will then produce a publicize copy and manage the dissemination at that time. The attending physicians at the hospital’s PD ward will receive the study’s details, findings, and directions on implementation if they wish to utilize it with their patients. Data sharing arrangements can be made upon reasonable request.

## Discussion

Explicit and cognitive-controlled timing abilities in Patients with PD are hypothesized to be impaired and manifest as a lack of motor-control seen in the hallmark tremors and gait symptoms. Contrarily, the implicit and automatic systems are thought to be still intact and can be engaged by using an external sensory stimulus (e.g., visual or auditory) implemented in interventions such as music therapy. These types of therapies have been shown to be effective for motor-timing rehabilitation especially in Patients with PD; however, benefits of such external sensory stimuli may be less effective for late staged patients with PD who have minimal motor control [43].

Many studies have measured the neural function in normal individuals using fNIRS but limited research to date has investigated and compared the changes in neural activity specifically in PD models. This is perhaps due to the inherent issues that arise from the severity of the patient’s tremor which include motion artifacts and detection of a superficial fNIRS hemodynamic response. To address such errors, additional correction factors and pre-signal processing prior to data analysis may be required.

With reporting and sharing the study’s findings, the intervention and conclusions drawn may also impact clinical practices and contribute to the PD research community. In particular, the results from this study can be used to encourage and inform the integration of music therapy to enhance PD treatment by supplementing or augmenting current practices with a similar activity. Implementation of a simple daily rehabilitation exercise may be prescribed especially for patients with early signs of PD to either maintain or improve motor control. Alternatively, larger studies with varying demographics may be explored to optimize the protocol. Furthermore, this study adds to the growing literature investigating the use of music therapy for patients with PD. More specifically, its novel use of fNIRS furthers the field’s neurological understanding of how PD impacts neuronal pathways and its recovery as well as the neurophysiological effects of sensorimotor movement in patients with PD. The locomotive freedom from an fNIRS setup allows for additional and more complex studies measuring the neuronal activation and trajectory of how music therapy using walking or larger motor movements impact patients with PD.

## Trial status

The present protocol is Version 1.1 completed on September 1, 2020. Recruitment for the study will begin January 2021 and continue for 18 months or until the required sample size is reached. Recruitment will be completed no later than July 31, 2022.

## Abbreviations

BR: Baseline rest
CFT: Continuous finger tapping
DBS: Deep brain stimulation
fMRI: Functional magnetic resonance imaging
fNIRS: Functional near-infrared spectroscopy
HbO_2_: Oxygenated hemoglobin
HbR: Deoxygenated hemoglobin
HC: Healthy control
MDS: Movement disorder society
MoCA: Montreal cognitive assessment
PD: Parkinson’s disease
SFT: Synchronous finger tapping
SMA: Supplementary motor area
SN: Substantia nigra
UPDRS: Unified Parkinson’s Disease Rating Scale
